# Time trends in treatment modes of anorexia nervosa in a nationwide cohort with free and equal access to treatment

**DOI:** 10.1002/eat.23378

**Published:** 2020-09-07

**Authors:** René Klinkby Støving, Pia Veldt Larsen, Laura Al‐Dakhiel Winkler, Niels Bilenberg, Michael Ejnar Røder, Hans‐Christoph Steinhausen

**Affiliations:** ^1^ Center for Eating Disorders Odense University Hospital Odense Denmark; ^2^ Research Unit for Medical Endocrinology Odense University Hospital Odense Denmark; ^3^ Department of Child and Adolescent Mental Health Odense Mental Health Services in the Region of Southern Denmark Odense Denmark; ^4^ Open Patient data Explorative Network (OPEN) Clinical Institute, University of Southern Denmark Odense Denmark; ^5^ Steno Diabetes Center Odense Odense University Hospital Odense Denmark; ^6^ Denmark Child and Adolescent Mental Health Centre Capital Region Psychiatry Copenhagen Denmark; ^7^ Department of Child and Adolescent Psychiatry Psychiatric University Hospital of Zurich Zurich Switzerland; ^8^ Clinical Psychology and Epidemiology Institute of Psychology, University of Basel Basel Switzerland

**Keywords:** age of onset, anorexia nervosa, hospitalization, incomes, outpatients, trends

## Abstract

**Background:**

Treating patients with anorexia nervosa (AN) remains a major challenge. The choice between an inpatient or an outpatient care setting is an essential issue for the patients and for their relatives with major health economic implications. However, health services‐related studies are lacking. The present study was a descriptive exploration of time‐trends in treatment modes of patients with free and equal access to health services.

**Methods:**

The study was based on a nationwide cohort of patients diagnosed for the first time with AN, each followed for 5 years in the registers covering the years 1994–2018. The per patient number of hospital admissions, cumulated number of days of hospitalization and number of outpatient visits during the first 5 years after initial diagnosis were considered.

**Results:**

The cohort of patients with AN with at least 5 years of follow‐up amounted to *N* = 7,505. A clear trend was observed in the per patient five‐year cumulated number of inpatient days, decreasing by 6% per year after adjustment for age at diagnosis, parental mental diagnosis, and family income. The five‐year number of hospital admissions after initial diagnosis decreased by 2% per year, while no trend was observed for outpatient visits.

**Conclusions:**

The per patient number of hospitalizations and cumulated days of hospitalization during 5 years after diagnosis were reduced for patients initially diagnosed with AN while there was no change in the number of outpatient visits. The factors contributing to these changes of treatment modes over time are in need of further study.

## 1 INTRODUCTION

The severity of anorexia nervosa (AN) varies from mild subclinical cases to chronic enduring and fatal incidents. The diagnostic criteria were revised in 2013 for the Fifth Edition of the *Diagnostic and Statistical Manual* (American Psychiatric Association, 2013) leading to markedly increased diagnostic heterogeneity and a 60% increase in lifetime prevalence (Mustelin et al., 2016), which is estimated to vary between 1 and 4% (Keski‐Rahkonen & Mustelin, 2016). Long‐term observations across 16 years between 1995 and 2010 based on Danish registry data indicated that the incidence of AN has increased from 6.4 to 12.6 per 100,000 person years (Steinhausen & Jensen, 2015).

The severe cases with AN may be regarded as intractable, and there is no evidence that the prognosis generally has improved substantially throughout the 20th century (Murray, Quintana, Loeb, Griffiths, & Le Grange, 2019; Steinhausen, 2002) with less than half of the treated patients achieving complete remission. However, mortality rates appear to decrease with specialized treatment (Winkler, Bilenberg, Horder, & Stoving, 2015). In clinical practice, the most severe cases may have been treated with long‐term hospitalizations lasting in some cases for several years.

There are differences in guideline recommendations regarding the preference of treatment modes. While the practice parameters of the American Academy of Child and Adolescent Psychiatry recommend outpatient care as first‐line treatment for children and adolescents with AN (Lock, La Via, American Academy of Child and Adolescent Psychiatry (AACAP) Committee on Quality Issues (CQI), 2015), the American Psychiatric Association advises outpatient care (for adults) only when the patient is highly motivated with a short duration of disease and a supportive family (American Psychiatric Association, 2013). However, the German national guidelines suggest treatment in hospital to continue until weight is fully restored (Herpertz et al., 2011). Moreover, the lengths of hospitalizations in Europe are reported to be considerably longer than in the United States (Madden et al., 2015), possibly due to economic issues, differences in insurance system, and varying access to treatment.

Although hospitalization for acute medical complications is a necessity to ensure survival, the benefits of further hospitalization remain questionable unless there are suicidal ideations, severe self‐harm, or intolerable family situations with intensive request. Prolonged hospitalization may have numerous profound negative consequences including loss of social networks and interruption of education or job. It is not known whether patients with AN in particular may be vulnerable to the negative effects of hospitalization.

The determinants and effects of hospitalization of patients with AN have been addressed in several studies. In a study of 1,185 consecutively admitted eating disorder patients in the United States the length of stay decreased from 150 days to 50 days from 1984 to 1998 with a marked increase in re‐admissions and decrease in discharge weight which led the authors to conclude that for some patients this change had been deleterious and not cost‐effective (Wiseman, Sunday, Klapper, Harris, & Halmi, 2001). Another study of 300 consecutive admissions in France found that duration of disease and the use of tube feeding were the strongest determinants of long‐term hospitalization (Strik Lievers et al., 2009). In a follow up study (*n* = 75) it was observed that hospital admission was a major predictor of poor outcome (Gowers, Weetman, Shore, Hossain, & Elvins, 2000). In a retrospective study of hospital charts, it was found that increased length of stay at hospital predicted rehospitalization (Willer, Thuras, & Crow, 2005). However, conclusions about the effect of long‐term versus short‐term hospitalization can hardly be drawn from observational studies.

Further correlates and potential determinants of hospitalization include age and sex of the patients (Favaro et al., 2009). Whereas adolescent age at onset of AN has been shown to have a favorable effect on the long‐term outcome of the disorder (Dobrescu et al., 2019; Steinhausen, 2002; Wentz, Gillberg, Anckarsater, Gillberg, & Rastam, 2009) and older age is less beneficial (Fichter, Quadflieg, Crosby, & Koch, 2017), there is no clear evidence on whether or not age at diagnosis has an impact on frequencies and durations of inpatient and outpatient care. There is some limited evidence that sex may be a relevant determinant since a study based on US‐American health insurance data showed for a one‐year period that females with AN received more days of treatment than their male counterparts (Striegel‐Moore, Leslie, Petrill, Garvin, & Rosenheck, 2000). This was in contrast to a smaller Danish clinical cohort study were it was found that males with AN were hospitalized more frequently than females with AN (Støving, Andries, Brixen, Bilenberg, & Hørder, 2011). In addition, the occurrence as well as the course of several mental disorders have been shown to be affected by parental income with elevated risks of eating disorders for children in higher income categories (Bjorkenstam et al., 2017). However, the potential influence of parental income on the different treatment modes in AN has not been investigated previously. Another relevant factor may be parental mental illness because besides increased familial aggregation of AN (Steinhausen, Jakobsen, Helenius, Munk‐Jørgensen, & Strober, 2015) any mental disorder may represent a factor of vulnerability and unfavorable parental support that may not only affect health care utilization of adolescents as shown recently for parental depression (Dreyer et al, 2018), but also the severity and treatment needs in AN.

There has been an increasing interest in comparing advantages and disadvantages of various treatment modes. In a Cochrane review of four trials including a total of 511 patients there was no clinically relevant difference between specialist inpatient care and outpatient care in weight gain at 12 months after start of treatment (Hay et al., 2019). The same conclusion was drawn from a recent observational study, where the AN outcome of long‐term strictly structured hospitalization was not superior to the outcome of a shorter stay with progressively increased patient autonomy around meal management without external incentives (Paquin Hodge et al., 2019). Long‐term stays may lead to fewer admissions, simply because re‐admission is possible only when the patient has been discharged. Furthermore, following long‐term hospitalization the risk for additional admission may either be lower because of effective treatment, or it may a priori be higher due to higher disease severity and a possible negative hospitalization effect with increased dependency including loss of social skills and network. That is, long‐term admissions may either protect against or may forward re‐admissions. Given the potential negative social effects of hospitalization, the total burden of hospitalization within a given period may be more important than the number of hospitalizations or the length of each hospitalization.

The aim of the present study was to explore time trends in different treatment patterns in terms of the per patient number of hospital admissions, per patient cumulated number of days of hospitalization, and per patient number of outpatient visits during the first 5 years after initial AN diagnosis in a nationwide large cohort of patients with AN in a setting with free and equal access to treatment in a public health system. The analyses were performed with an adjustment for age at diagnosis, sex, family income, and parental mental diagnosis.

## 2 METHODS

### 2.1 Study sample

The study comprises a nationwide cohort of patients diagnosed for the first time with AN (F50.0) or atypical AN (F50.1) according to the *International Statistical Classification of Diseases and Related Health Problems*, 10th edition (ICD‐10) (WHO, 1992) in the Danish National Patient Register (NPR) in the period 1994–2013. Atypical AN is defined as a disorder meeting some criteria of the typical form (BMI below 17.5 kg/m2, self‐induced weight loss, body image distortion, and endocrine disorder most often reflected by secondary amenorrhea) without fulfilling all key symptoms. All patients with a previous eating disorder diagnosis (F50x [ICD‐10] or 305.60, 306.50, 306.58, 306.59 [ICD‐8]) were excluded.

**FIGURE 1:**
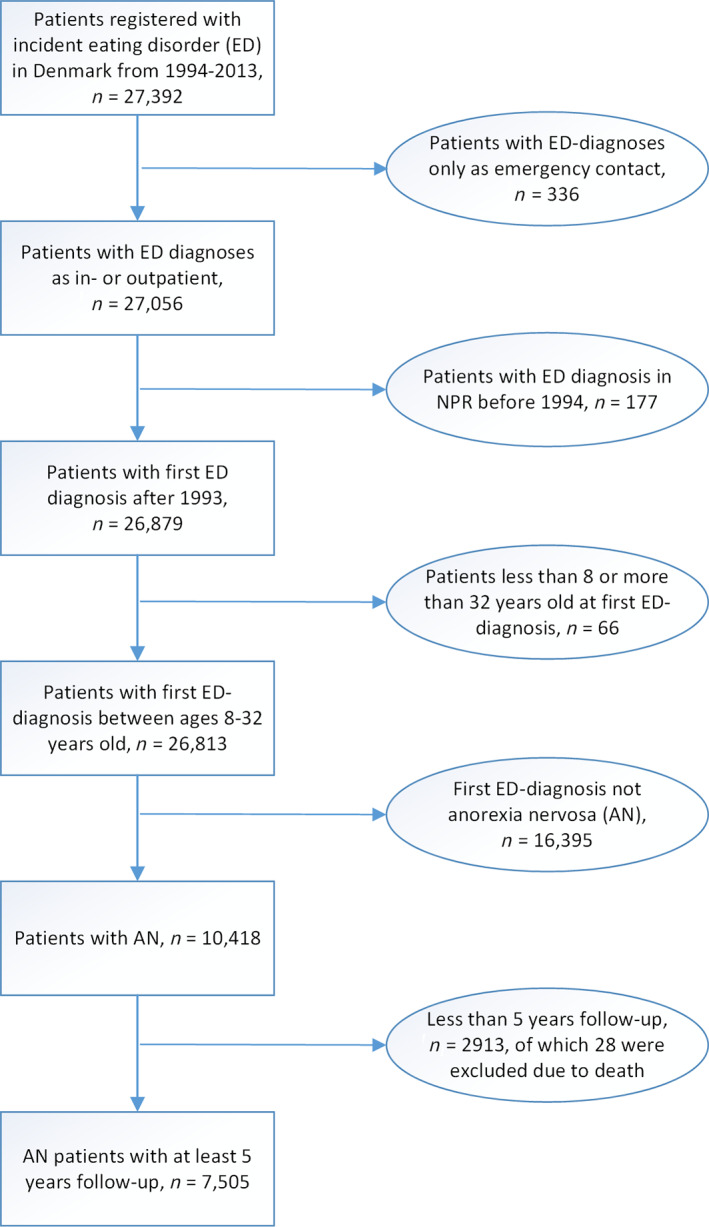
Flow chart for identifying the final study population, *n* = 27,392 [Color figure can be viewed at wileyonlinelibrary.com]

Each patient in the study sample was followed for 5 years after diagnosis in the registers covering the years 1994–2018 in total. The first 5 years of treatment were chosen to minimize the effect of enduring long‐standing courses with recoveries and relapses, which change the approach as described in a recent review (Russell, Mulvey, Bennett, Donnelly, & Frig, 2019) and which may blur potential trends. All patients with less than a 5‐year follow‐up were excluded; the same applied to patients who were included less than 5 years before the end of the study period 31/12–2018 (Figure 1). The age range at first time diagnosis was set at a minimum of 8 years and the empirical maximum was 32 years. Patients with an AN‐diagnosis before age 8 were excluded due to unclear validity of the diagnosis given the major difficulties of differential diagnosis at this young age.

The sample of patients with AN (F 50.0) or atypical AN (F50.1) with at least 5 years follow‐up amounted to *N* = 7,505 (Figure 1). All patients were hospitalized as either in‐ or outpatient at the time of the incident AN diagnosis in the NPR. Because the number of day‐patient treatments were very few (<0.50%) they were regarded as inpatient treatments. A total of 1,326 (17.7%) of the patients with AN in the final study population were initially diagnosed in an inpatient setting, while 6,179 (82.3%) were diagnosed in an outpatient setting. Emergency room contacts were included only if they had a subsequent hospitalization because of doubts in the validity of diagnosis in cases with an emergency contact only. Overlapping hospitalizations, for example, somatic supervision of a patient hospitalized on a psychiatric ward, were merged (combined into one admission) if they overlapped by at least 1 day, including the date of admission and the date of discharge, before calculating the number of hospitalizations.

### 2.2 Procedure

The study was based on the comprehensive Danish system of registries with mandatory recording of each patient visit to the public health service, covering various domains of data including extensive health information on the total national population (Pedersen, 2011). In the present study, based on the personal identification number (CPR) assigned to all Danish citizens and residents, information on diagnoses and in‐ and outpatient treatments was collected from the NPR; date of birth, sex, postcode of residence from the CPR register; and family income from the income register at the time of the first AN diagnosis in the NPR. Data were provided by Statistics Denmark in an anonymized fashion and approval of the study was given by the Danish Data Protection Agency (file no. 15/280490). According to Danish law, ethical approval is not required for registry‐based studies. Access to the data requires application to the Danish authorities.

### 2.3 Statistical analyses

Patients registered with a first‐ever AN diagnosis in the NPR in the period 1994–2013 were included if they had at least 5 years of follow‐up in the registers after the initial diagnosis. The linear association between calendar year and the proportion of incident AN patients relative to the Danish background population in the same age interval was analyzed using linear regression. Residual analyses indicated no deviations from the model assumptions. For each patient, data from the first 5 years after diagnosis were used in order to calculate the patient’s number of hospital admissions, cumulated number of days of hospitalization and number of outpatient visits during the first 5 years after initial AN diagnosis. For example, a patient included in 2013 was followed in the period 2013–2018. Patients with less than 5 years of follow‐up (whether due to the end of the study period on December 31, 2018 or death) were excluded (Figure 1), since the three 5‐year treatment outcomes could not be calculated for these patients.

Descriptive statistics on patient characteristics at the time of inclusion on sex, age, family income (in tertiles by calendar year) and previous mental disorders of parents (none, ≥ 1 diagnosis in a single parent, or ≥ 1 diagnosis in both parents; according to diagnosis codes DFxx [ICD‐10] or 290xx‐309xx [ICD‐8] in the NPR from 1976) were computed for all years combined and separately by calendar year. Further, for each calendar year, the mean, standard deviation, median and interquartile range were calculated for each of the three 5‐year treatment outcomes, that is, the per patient number of hospital admissions, per patient cumulated number of days of hospitalization and per patient number of outpatient visits during the first 5 years after initial AN diagnosis. Spearman correlation was used to calculate pairwise correlations between the overall annual means of the three 5‐year treatment outcomes from 1994–2013.

Associations in terms of incidence rate ratios (IRRs) between each of the three patient‐level 5‐year treatment outcomes and age (in years), sex, family income (in tertiles by calendar year) at the time of inclusion, and parental mental disorder prior to inclusion (none, or at least one diagnosis in at least one parent) were analyzed using negative binomial regression with robust standard errors, mutually adjusting for sex, age, income tertile, parental mental disorder prior to inclusion, and calendar year. Negative binomial distributions were used instead of Poisson distributions to address overdispersion in the data. Family income was treated as a categorical variable in the analyses due to residual analyses indicating broken model assumptions on linearity of income as continuous variables in the regression models.

The crude and adjusted time trends from 1994 to 2013 of each of the patient‐level 5‐year treatment outcomes were analyzed in terms of IRRs using negative binomial regression with robust standard errors and calendar year of first‐time AN diagnosis as independent variable, adjusting for sex, age, and income tertile at the time of inclusion, and parental mental disorder prior to inclusion. The adjusted time trends of each of the three patient‐level 5‐year treatment outcomes were illustrated graphically as marginal (by year) predicted means with 95% confidence intervals (CIs) based on negative binomial regression with robust standard errors.

## 3 RESULTS

Sample characteristics are shown in Table 1. Among *N* = 7,505 patients with first‐time AN diagnoses in 1994–2013, 94% were females and the average age at diagnosis was 18.0 years. In some 19% of the AN patients one or two parents had received the diagnosis of a mental disorder. The average age of patients with first‐time AN diagnosis decreased from 18.4 to 17.6 years from 1994 to 2013 (Supplementary Table 1). The number of incident patients with AN who sought treatment in the mental care system increased from 153 in 1994 to 654 in 2013 (Table 2 and Supplementary Table 1, test for linear trend was significant with *p* < .001).

**TABLE 1:** Characteristics patients with first‐time diagnosis of AN in 1994 – 2013^a^, *n* = 7,505

	All patients
Total, *n*	7,505
Sex	
Female, *n*(%)	7,028 (93.6)
Male, *n*(%)	477 (6.4)
Age in years, mean (*SD*) [range]	18.0 (4.9) [8–32]
Family income group (tertiles by year)	
Lower tertile, *n*(%)	2,504 (33.4)
Middle tertile, *n*(%)	2,497 (33.3)
Higher tertile, *n*(%)	2,488 (33.2)
Previous mental disorders of parents^b^	
None, *n*(%)	6,084 (81.1)
≥1 diagnoses in single parent, *n*(%)	1,253 (16.7)
≥1 diagnoses in both parents, *n*(%)	168 (2.2)

^a^ Characteristics of incident AN patients by year are shown in Supplementary Table 1.

^b^ Any mental diagnosis in parent since 1976 (including eating).

**TABLE 2:** Per patient number of hospital admissions, cumulated number days of hospitalization and number of outpatient visits during 5 years follow‐up after first‐time AN diagnosis, by calendar year of first‐time AN diagnosis for the period 1994–2013, *n* = 7,505

		Per patient number of hospital admissions during 5 years follow‐up after diagnosis		Per patient cumulated days of hospitalization during 5 years follow‐up after diagnosis		Per patient number of outpatient visits during 5 years follow‐up after diagnosis	
Year of first‐time AN diagnosis	Number of incident AN patients	Mean (*SD*)	Median [IQR]	Mean (*SD*)	Median [IQR]	Mean (*SD*)	Median [IRQ]
1994	153	3.73 (4.76)	2 [4]	169.65 (331.74)	27 [127]	7.08 (12.42)	2 [10]
1995	259	2.85 (4.31)	2 [4]	96.64 (208.92)	10 [100]	4.78 (8.93)	2 [6]
1996	206	3.04 (4.78)	2 [4]	105.20 (205.52)	18.5 [109]	7.37 (23.88)	2 [7]
1997	235	2.91 (4.06)	2 [4]	116.70 (234.46)	13 [108]	5.70 (11.77)	1 [6]
1998	313	2.71 (4.05)	2 [3]	115.66 (260.83)	14 [82]	6.50 (11.40)	2 [8]
1999	339	2.46 (3.67)	1 [3]	88.47 (218.08)	7 [65]	9.99 (34.17)	3 [10]
2000	342	2.63 (3.84)	1 [4]	79.82 (180.84)	9.5 [79]	8.70 (17.36)	2.5 [9]
2001	306	2.16 (3.57)	1 [3]	68.75 (166.19)	4 [40]	8.18 (15.53)	3 [10]
2002	351	2.28 (3.34)	1 [3]	87.76 (198.42)	9 [91]	9.04 (17.36)	3 [10]
2003	350	2.40 (4.00)	1 [3]	87.17 (204.38)	6 [71]	8.45 (14.73)	3 [10]
2004	335	2.25 (6.12)	1 [2]	71.81 (168.70)	4 [43]	6.91 (11.46)	3 [9]
2005	375	2.08 (3.75)	1 [3]	53.89 (140.53)	2 [25]	8.05 (13.30)	3 [8]
2006	379	2.21 (4.51)	1 [2]	63.79 (144.74)	3 [54]	7.92 (12.87)	4 [9]
2007	412	2.09 (3.41)	1 [3]	61.71 (145.16)	3 [52.5]	8.74 (13.92)	4 [10]
2008	453	2.02 (3.22)	1 [2]	57.02 (153.73)	4 [34]	9.18 (14.20)	4 [10]
2009	463	2.21 (4.37)	1 [3]	58.08 (151.48)	3 [28]	9.42 (14.76)	4 [11]
2010	484	2.07 (4.42)	1 [3]	43.25 (108.18)	2 [30.5]	6.93 (12.74)	3 [7.5]
2011	532	2.35 (7.58)	1 [2]	53.35 (154.58)	2 [20]	7.57 (11.87)	3 [8]
2012	564	1.86 (3.81)	1 [2]	42.98 (121.03)	2 [18.5]	8.09 (14.09)	3 [8]
2013	654	1.90 (5.80)	1 [2]	37.03 (109.99)	1 [12]	6.90 (11.74)	2 [7]

Abbreviations: *SD*, standard deviation; IQR, interquartile range.

The per patient mean number of hospital admissions, per patient cumulated number of days of hospitalization and per patient number of outpatient visits during the first 5 years after first‐time AN diagnosis are displayed in Table 2 for each calendar year of initial AN diagnosis 1994 to 2013. Across the years of initial AN diagnosis, 1994–2013, the per patient mean number of hospitalizations during 5 years after initial diagnosis varied between 1.8 to 3.7 admissions, the cumulated number of inpatient days varied between 37 to 169, and the number of outpatient visits varied between 5.7 to 10.0 (Table 2). A total of 2,606 patients (34.7%) had no hospital admissions, and thus no hospitalization days, while 1985 patients (26.5%) had no outpatient visits during the 5 years of follow‐up.

The overall annual means of per patient number of hospital admissions and per patient cumulated number of days of hospitalization during the first 5 years after initial AN diagnosis were highly correlated (r_*s*_ = 0.91, *p* < .001), while neither was correlated with per patient number of outpatient visits (both *p* > .2). Supplementary Figure 1 shows, for each of the three per patient five‐year treatment outcomes, the adjusted marginal (by year of initial diagnosis 1994–2013) predicted means with 95%‐CIs, adjusted for age at diagnosis, sex, family income tertile, and parental mental disorder prior to inclusion. A clear trend was observed in the per patient five‐year cumulated number of inpatient days, decreasing by 6% per year across the years of initial diagnosis (crude IRR (95%‐CI) = 0.94 (0.93, 0.95), adjusted IRR (95%‐CI) = 0.94 (0.93, 0.95)). The per patient five‐year number of hospital admissions after initial diagnosis decreased by 2% per year (crude IRR (95%‐CI) = 0.98 (0.97, 0.99), adjusted IRR (95%‐CI) = 0.98 (0.97, 0.98)), while no significant trend was observed for per patient five‐year number of outpatient visits (crude IRR (95%‐CI) = 1.00 (1.00, 1.01), adjusted IRR (95%‐CI) = 1.00 (0.99, 1.01)).

Age at diagnosis was associated with per patient five‐year cumulated number of inpatient days (IRR (95%‐CI) per year of age = 0.97 (0.96, 0.98), adjusted for sex, income tertile, parental mental diagnosis, and calendar year), and five‐year number of outpatient visits (IRR (95%‐CI) = 1.04 (1.04, 1.05)), but not with per patient five‐year number of hospital admissions. Female AN patients had more hospital admissions per patient than males during 5 years after initial AN diagnosis (IRR (95%‐CI) = 1.36 (1.16, 1.59), adjusted for age, income, parental mental diagnosis, and calendar year), but no association was observed between sex and per patient five‐year numbers of outpatient visits or per patient five‐year cumulated length of stay in hospital.

The family income (in tertiles by year) was associated with per patient number of hospital admissions (reference lower income tertile: IRR (95%‐CI) for middle income tertile = 0.89 (0.80, 1.00), IRR (95%‐CI) for higher income tertile = 0.79 (0.70, 0.89), adjusted for sex, age, parental mental diagnosis, and calendar year), but not with per patient number of outpatient visits or per patient cumulated length of stay in hospital. Parental mental diagnosis at baseline was associated with per patient number of outpatient visits (IRR (95%‐CI) = 1.15 (1.04, 1.27), adjusted for sex, age, income, and calendar year), and per patient number of hospital admissions (IRR (95%‐CI) = 1.31 (1.16, 1.48)), and marginally with per patient cumulated length of stay in hospital (IRR (95%‐CI) = 1.16 (1.00, 1.34).

## 4 DISCUSSION

The major finding of this study was a clear time‐trend at the patient level for patients with first‐time AN diagnosis in 1994–2013 of decreasing cumulative number of inpatient days and decreasing number of hospital admissions during 5 years after initial AN diagnosis. These trends were robust after adjustments for age at diagnosis, sex, family income, and parental mental disorder.

During the observation period, inpatient treatment may have covered different therapeutic programs changing over time. However, all treatment had been carried out in public hospitals subjected to guidelines from the national health board ensuring uniformity and an evidence‐based approach (National Board of Health, 2016). Contrary to a previous study (Wiseman et al., 2001), the marked reduction in cumulated length of hospitalization in the present study was not reflected in an increase in the number of hospital admissions. From a clinical point of view, preparation of discharge and aftercare is crucial, but drop‐out and nonadherence is a recognized feature of the syndrome. Unfortunately, indicators of the severity of the disorder such as low BMI, low weight gain during treatment, and purging behavior as predictors for re‐admission (Steinhausen, Grigoroiu‐Serbanescu, Boyadjieva, Neumarker, & Winkler Metzke, 2008) are not registered in the NPR, which prevents analyzing associations between severity and hospitalizations in the present study.

Notably, the data of the present study do not provide evidence that reduction in length of hospitalizations leads to a higher frequency of admission. This is important since brief stays for stabilization a priori may be associated with less stigma, and improved maintenance of usual social‐ and work activities. Though the present study does not include any information on longterm outcome of AN, it should be considered that controlled trials have not provided evidence of a better outcome in favour of hospitalization (Hay et al., 2019). Moreover, there is evidence, that the mortality in AN has declined during the last decades (Lindblad, Lindberg, & Hjern, 2006; Winkler et al., 2015). Obviously, controlled studies are required to determine the optimal treatment setting tailored to the stage of illness (Hay et al., 2019), especially studies that include patient reported outcome data (Winkler et al., 2017). In addition to the length of hospitalization there are numerous factors which may affect long term outcome in AN, and no consensous exists to clearly define remision and recovery. It is beyond the scope of this study to analyze associations between patient treatment patterns and other patient outcomes.

Our findings confirm a considerable increase in incident patients with AN who sought treatment in the mental care system over the 20 years of the observation period. This increasing tendency applies to psychiatric disorders in general, and it has previously been shown in a Danish registry study that the increase in patients seeking treatment for AN is at the same level as for other psychiatric disorders (Steinhausen & Jensen, 2015). We found no clear association between age at diagnosis and the per patient number of hospital admissions during 5 years after initial AN diagnosis. However, the per patient cumulated length of hospitalization in the 5 years following first‐time diagnosis decreased with increasing age. This may have been motivated by the conviction that early intervention in adolescents may avoid chronification of the disorder (Zipfel, Giel, Bulik, Hay, & Schmidt, 2015).

We found that male AN patients had significantly less hospital admissions per patient than females during 5 years after initial AN diagnosis. This sex difference is in line with a previous study (Striegel‐Moore et al., 2000). Furthermore, patients from a higher family income background had a lower number of inpatient admissions which could be due to a higher adherence to outpatient treatment compared to families with lower income. As expected, the presence of a parental mental disorder was significantly associated with higher numbers of both inpatient and outpatient admissions. Negative effects of the stresses imposed by a parental mental disorder on the offspring and an effect of health‐care utilization as shown recently for parental depression (Dreyer et al., 2018) may have been operant. Our observational study does not allow any causal conclusions about the definite factors contributing to decreased hospitalization. Future studies may consider hypotheses on associations between AN outcomes and covariates over time.

In parallel to a major trend of economic pressure on the health system, decreased hospitalization might have been driven by a wish to reach sizeable cost savings. Indeed, in a study of a three‐month treatment—which did not include patients with BMI < 15—the largest share of costs resulted from hospitalizations (Stuhldreher et al., 2015). However, appropriate comparative economic studies of the total costs of different modes of treating AN are very sparse (Stuhldreher et al., 2012). Furthermore, the reduced hospitalization might be a result of changed clinical practice with higher threshold for admission. However, during the whole period of the present study, outpatient treatment consistently has been recommended as first line of treatment, and no alterations in admission criteria are expressed in our national authoritative guidelines 2016 (National Board of Health, 2016) compared to 2005 (National Board of Health, 2005). Last but not least, it cannot be ruled out that inpatient treatment in general gradually has become more effective in parallel with a tendency towards increasing specialization and/or the capacity to support patients in a medically safe manner in outpatient settings may have been improved as well.

The present study is based on a large nationwide sample and long‐term follow‐up in a country that is homogeneous regarding sociodemographics and access to tax‐funded healthcare. The diagnostic classification is based solely on ICD‐10 criteria during the whole period of inclusion 1994–2013 and, thus, is not affected by the essential shift from DSM‐IV to DSM‐5. However, there are limitations to acknowledge. First, the registers only include treatment seeking patients. Thus, less severe cases of AN may have remained undetected, and lack of recognition of symptoms among patients with AN may have contributed to selection bias which is a general challenge in studies of this group of patients. Nevertheless, free access without costs to the public health system and a clearly defined responsibility of care for these patients by specialists rather than general practitioners may have minimized the selection bias. Secondly, the Danish registers do not cover patients who seek private financed treatment. However, since specialized treatment in the public health system is free and accessible, the private care constitutes only a very small part in the Danish health provision system (Ministry of Health, Denmark, 2017). Thirdly, there is no scientific validation of the accuracy of diagnoses entered in the registers, although quality checks suggest that the diagnostic validity is high across a range of disorders (Mohr‐Jensen, Vinkel Koch, Briciet Lauritsen, & Steinhausen, 2016). Fourthly, exclusion of patients who died during the 5‐year follow‐up period could lead to bias since they may be more severe cases. However, as only 28 patients in total (0.27%) were excluded due to death, this bias is likely to be negligible. Fifthly, the per patient observation period was restricted to 5 years from diagnosis to ensure that recent cases had the same opportunity to be followed as cases in the early study years. Given the lack of consensus for the definition of the severe and enduring stage (Hay & Touyz, 2018), we decided to exclude treatment activities beyond the first 5 years. Further, it is likely that severity of AN varies among the included patients at the time of their incident diagnosis. However, the NPR does not provide information on severity of the disease. Finally, the pairwise Spearman correlations between the overall annual means of the three 5‐year treatment outcomes may be affected by aggregation bias and should be interpreted with caution.

In conclusion, the patient‐level cumulated length of hospitalizations during 5 years after initial AN diagnosis has been reduced dramatically along with a decrease in hospital admissions for Danish patients with first‐time AN diagnosis in the period 1994–2013. However, our observational study does not allow for causal conclusions and clinical recommendations regarding treatment modes. For this purpose, we recommend controlled intervention studies to examine outcomes of AN after short versus long‐term hospitalizations.

## CONFLICT OF INTEREST

The authors have no conflict of interest.

## Supporting information


**Appendix** **S1**: Supporting information

## Data Availability

The data that support the findings of this study are available by application to Statistics Denmark (https://www.dst.dk/en).
