# Supplementary material for: Time trends in treatment modes of anorexia nervosa in a nationwide cohort with free and equal access to treatment
Source: Int J Eat Disord. 2020 Sep 7;53(12):1952–9. doi: 10.1002/eat.23378 (PMC7754480; doi:10.1002/eat.23378)
Supplement: Supplementary file 1 — Appendix S1: Supporting information [file EAT-53-1952-s001.docx]

## **Supplementary Table 1. Characteristics patients with first-time diagnosis of AN in 1994 – 2013, by calendar year of first-time AN diagnosis for the period 1994-2013, n = 7505**

|  | **1994** | **1995** | **1996** | **1997** | **1998** | **1999** | **2000** | **2001** | **2002** | **2003** | **2004** | **2005** | **2006** | **2007** | **2008** | **2009** | **2010** | **2011** | **2012** | **2013** |
| --- | --- | --- | --- | --- | --- | --- | --- | --- | --- | --- | --- | --- | --- | --- | --- | --- | --- | --- | --- | --- |
| **Total, n** | 153 | 259 | 206 | 235 | 313 | 339 | 342 | 306 | 351 | 350 | 335 | 375 | 379 | 412 | 453 | 463 | 484 | 532 | 564 | 654 |
| **Sex** |  |  |  |  |  |  |  |  |  |  |  |  |  |  |  |  |  |  |  |  |
| Female, n(%) | 148 (96) | 241 (93) | 198 (96) | 225 (96) | 299 (96) | 325 (96) | 323 (94) | 291 (95) | 327 (93) | 328 (94) | 310 (93) | 349 (93) | 352 (93) | 382 (93) | 430 (95) | 426 (92) | 450 (92) | 483 (91) | 525 (93) | 616 (94) |
| Male, n(%) | 5 (4) | 18 (7) | 8 (4) | 10 (4) | 14 (4) | 14 (4) | 19 (6) | 15 (5) | 24 (7) | 22 (6) | 25 (7) | 26 (7) | 26 (7) | 30 (7) | 23 (5) | 37 (8) | 37 (8) | 49 (9) | 39 (7) | 38 (6) |
| **Age in years, mean (SD)** | 18.4 (4.9) | 18.5 (4.8) | 18.1 (4.9) | 18.6 (4.8) | 18.8 (5.1) | 18.8 (5.0) | 19.1 (5.1) | 17.8 (4.9) | 17.8 (4.9) | 18.0 (5.1) | 18.1 (5.1) | 18.1 (5.4) | 17.7 (5.0) | 17.7 (5.0) | 17.9 (4.7) | 17.6 (4.8) | 17.3 (4.6) | 17.5 (4.7) | 17.7 (4.6) | 17.6 (4.5) |
| **Family income group (tertiles)** |  |  |  |  |  |  |  |  |  |  |  |  |  |  |  |  |  |  |  |  |
| Lower tertile, n(%) | 52 (34) | 86 (33) | 70 (34) | 75 (32) | 107 (34) | 114 (34) | 114 (33) | 103 (34) | 117 (33) | 116 (33) | 111 (33) | 125 (33) | 128 (34) | 137 (33) | 151 (33) | 154 (33) | 162 (34) | 175 (33) | 188 (33) | 219 (334) |
| Middle tertile, n(%) | 51 (33) | 86 (33) | 68 (33) | 80 (34) | 104 (33) | 112 (33) | 114 (33) | 101 (33) | 117 (33) | 117 (34) | 112 (33) | 125 (33) | 125 (33) | 138 (34) | 151 (33) | 154 (33) | 161 (33) | 176 (33) | 188 (33) | 217 (33) |
| Higher tertile, n(%) | 50 (33) | 87 (34) | 68 (33) | 79 (34) | 102 (33) | 112 (33) | 114 (33) | 102 (33) | 117 (33) | 115 (33) | 112 (33) | 125 (33) | 126 (33) | 136 (33) | 150 (33) | 154 (33) | 159 (33) | 177 (34) | 187 (33) | 216 (33) |
| **Previous mental disorders of parents^a^** |  |  |  |  |  |  |  |  |  |  |  |  |  |  |  |  |  |  |  |  |
| None, n(%) | 143 (93) | 238 (92) | 179 (87) | 206 (88) | 269 (86) | 295 (87) | 277  (81) | 256 (84) | 302 (86) | 297 (85) | 268 (80) | 298 (79) | 302 (80) | 328 (80) | 341 (75) | 359 (78) | 376 (78) | 419 (79) | 438 (78) | 493 (75) |
| ≥1 diagnoses in one or both parents, n(%) | 10 (7) | 21 (8) | 27 (13) | 29 (12) | 44 (14) | 44 (13) | 65 (19) | 50 (16) | 49 (14) | 53 (14) | 67 (20) | 77 (21) | 77 (20) | 84 (20) | 112 (25) | 104 (22) | 108 (22) | 113 (21) | 126 (22) | 161 (25) |

^a^Any mental diagnosis in parent since 1976 (including eating disorders) until date of inclusion.

**Supplementary Figure 1. Adjusteda marginal predicted means with 95-confidence intervals of patient-level number of hospital admissions (A), cumulated number of days of hospitalization (B) and number of outpatient visits (C) during five years follow-up after initial diagnosis for patients with first-time AN diagnosis in the period 1994-2013, n=7505.**
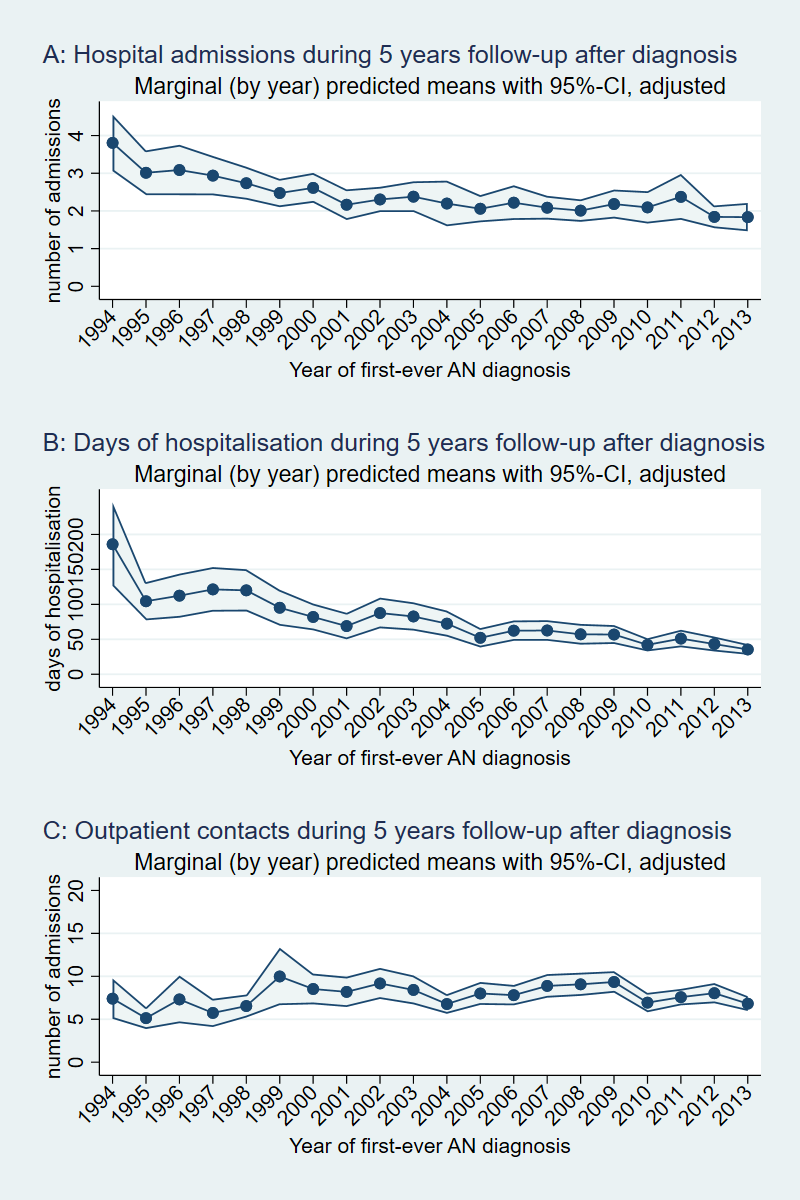


aAdjusted for patient sex and age in years, family income in tertiles (by year) and previous mental disorder in parents (none vs. at least one diagnosis in at least one parent), using negative binomial regression models with robust standard errors

Abbreviations: CI = confidence interval
